# A Conceptual Framework for Evaluation of Public Health and Primary Care System Performance in Iran

**DOI:** 10.5539/gjhs.v7n4p341

**Published:** 2015-01-25

**Authors:** Nader Jahanmehr, Arash Rashidian, Ardeshir Khosravi, Farshad Farzadfar, Mohammad Shariati, Reza Majdzadeh, Ali Akbari Sari, Alireza Mesdaghinia

**Affiliations:** 1 Department of Health Management and Economics, School of Public Health, Tehran University of Medical Sciences, Tehran, Iran; 2 Department of Global Health and Public Policy, School of Public Health, Tehran University of Medical Sciences, Tehran, Iran; 3 Knowledge Utilization Research Center, Tehran University of Medical Sciences, Tehran, Iran; 4 Center for Primary Health Care Management, Ministry of Health and Medical Education, Tehran, Iran; 5 Non-communicable Diseases Research Center, Tehran University of Medical Sciences, Tehran, Iran; 6 Endocrinology & Metabolism Population Sciences Institute, Tehran University of Medical Sciences, Tehran, Iran; 7 Department of Epidemiology and Biostatistics, School of Public Health, Tehran University of Medical Sciences, Tehran, Iran; 8 Department of Community Medicine, School of Medicine, Tehran University of Medical Sciences, Tehran, Iran; 9 Department of Environmental Health Engineering, School of Public Health, Center for Water Quality Research, Institute for Environmental Research, Tehran University of Medical Sciences, Tehran, Iran

**Keywords:** conceptual framework, health deputy, monitoring and evaluation, performance, results chain, health system, input, output, outcome

## Abstract

**Introduction:**

The main objective of this study was to design a conceptual framework, according to the policies and priorities of the ministry of health to evaluate provincial public health and primary care performance and to assess their share in the overall health impacts of the community.

**Methods:**

We used several tools and techniques, including system thinking, literature review to identify relevant attributes of health system performance framework and interview with the key stakeholders. The PubMed, Scopus, web of science, Google Scholar and two specialized databases of Persian language literature (IranMedex and SID) were searched using main terms and keywords. Following decision-making and collective agreement among the different stakeholders, 51 core indicators were chosen from among 602 obtained indicators in a four stage process, for monitoring and evaluation of Health Deputies.

**Results:**

We proposed a conceptual framework by identifying the performance area for Health Deputies between other determinants of health, as well as introducing a chain of results, for performance, consisting of Input, Process, Output and Outcome indicators. We also proposed 5 dimensions for measuring the performance of Health Deputies, consisting of efficiency, effectiveness, equity, access and improvement of health status.

**Conclusion:**

The proposed Conceptual Framework illustrates clearly the Health Deputies success in achieving best results and consequences of health in the country. Having the relative commitment of the ministry of health and Health Deputies at the University of Medical Sciences is essential for full implementation of this framework and providing the annual performance report.

## 1. Introduction

Performance measurement means efforts to monitor, evaluate and establish the relationship between the goals, resources and the activities within the organization, with the results, outputs and achievements of the desired goals (Smith, 2009). The health system is a complex system with different stakeholders, including patients, service providers, policy makers, service buyer organizations, the Government and the vast expanse of the citizens, and the Community (Smith, Mossalios, & Papanicolas, 2008). To achieve the objectives of the health system; all the stakeholders with a set of relationships can be associated with each other. The main role of the Monitoring and Evaluation (M&E) system, is to pay special attention to the performance of each of these stakeholders through informing them about their decisions and the results of their performance on the health system. For example, Governments and policy makers typically need to provide tools for monitoring and performance assessment of the health system, in order to decide on the optimal allocation of resources and carry out the necessary policies and interventions. Researchers for the production of scientific evidence in order to carry out reforms in the health system, and donor agencies to ensure that aid is effective, paying more attention to performance indicators and evaluation results (Kruk & Freedman, 2008).

Measuring and evaluating performance is one of the most important concerns of the health system in any country. Recent research results show that among developing countries with similar economic and educational conditions, there is a huge difference in health indicators and outputs. Part of this is due to the obvious difference in performance observed in different health systems (Murray & Frenk, 2000).

### 1.1 Iran’s Health System

Over the past three decades, Iran’s health system has made great achievements, with the help of codified and regular programs, particularly in the public health sector and Primary Health Care (PHC). Increased life expectancy, reduction of mothers and children’s mortality, significant reduction in the prevalence and incidence of communicable diseases, improved sanitation, safe drinking water supply, maximum coverage of services and expansion of the health network across the country, were only a part of Iran’s health system’s success in this period (Lankarani, Alavian, & Peymani, 2013; LeBaron & Schultz, 2005; World health statistics, 2014).

Iran, today has a vast network of PHC units and very good coverage in rural areas and cities. The family physician program is running in all rural areas and cities with under 20,000 residents since the second half of 2005 (Takian, Rashidian, & Kabir, 2011) and should be run for all the people of Iran based on the fifth development plan (2011). These changes help to improve the level of people’s health in Iran. Despite the important progress and success in the health system of Iran, for multiple reasons the problems of the current health system are considerable, with different challenges such as the change in the age structure within the population, increasing urbanization, changing lifestyle and increase in non-communicable diseases (Moghaddam et al., 2013). Based on the results from the current study, the economic cost of burden of disease has been important and will amount to about 10% of the country’s GDP.

Health system policy and planning usually takes place at the national level and is concentrated. Universities of medical sciences, are mostly executive policies and programs of the Ministry of Health and Medical Education (MOHME), and in spite of the decision-making being based upon local conditions in the province, many of the policies are run in the same way at the Universities. Compared with neighboring and developing countries in the past two decades, in Iran in accordance with international standards, and even beyond the country’s facilities, large national research in the field of demographic and health has been conducted. Important studies like DHS (Ministry of Health and Medical Education [MOHME], 2002), IrMIDHS (Rashidian et al., 2012), MICS (MOHME, 1997) and Utilization (MOHME, 2005) show this issue very well. In such studies, and especially in registered data collection, a huge volume of data has been collected, and despite spending a lot of resources and manpower, may not be used much in practice. A large part of the services that have been provided were solely based on the managers recognition of health needs within the community and rely less on information resources(Farzadfar, Haddadi, Nayeli, Moghimi, & Mollasheikhi, 2005). Measurement and evaluation of health programs are not complete and organized, and thus there is still much to do to create a comprehensive and integrated information system in the country.

### 1.2 Describing the Health Deputy

After the merger of medical education in the health system in 1985 (Azizi, 2009), the MOHME in Iran now has 56 universities and medical schools, the term University of Medical Sciences will be used for all of them in this study. The macro planning and policy making for these universities has been done by the MOHME. According to the current structure of the MOHME, all Universities of Medical Sciences have a Health Deputy as well as other deputies. Deputy of health at each University is responsible for first-level services, including public health and primary health care. Deputy of health in terms of the number of personnel and health service provider centers includes a wide area (Shirvani et al., 2011). All Health Deputies have the same structure and hierarchy and the majority of the population in all parts of the country is covered by the services they provide. All provinces have at least one University of Medical Sciences, some provinces, such as Esfahan and Fars have several Universities, with each of them solely providing the services for population they cover.

### 1.3 Performance Monitoring and evaluation of Health Deputy

With regard to the limitation of resources, Health Deputy’s administrators are constantly faced with these questions: what are the achievements of health programs for the society? Is it possible to attribute all the desired changes in the impact indicators of health in population to health system performance? For example, a measure like the Pediatric mortality, is considered in most performance assessments, but it is not clear what share of it is as a result of health system performance. Is it possible to say that the other determinants of health have no effect on health impact indicators? If the answer for these questions is negative, then what is the share of districts of health activities in the changes of health impact indicators? (Farzadfar et al., 2005).

A large part of the problems that were talked about, are due to lack of an integrated management information system (Fazaeli, Ahmadi, Rashidian, & Sadoughi, 2014) and lack of monitoring and evaluation in Iran’s health system. Monitoring and evaluation system, through the provision of regular performance reports, gives all data requirements to managers for planning and decision-making. The existence of this system can meet the needs of the organization and society, and indicate the effect of the activities and increase the system’s ability to respond.

Reviews on the Health Deputy of the MOHME and the results of interviews conducted during this study with experts in the health system, show past attempts to evaluate the performance of Health Deputies at the University of medical sciences, but this issue does not have continuity and has a lot of flaws and was given up. Usually, they are assessed by annual self-assessment (Shirvani et al., 2011).

In recent years, the subject of performance evaluation has been increasingly reflected in macro policies, at the MOHME and government level. Management information system (MIS) and monitoring and evaluation of health sector performance has been emphasized in “the fourth and fifth comprehensive development program” (Vice-Presidency for Strategic Planning and Supervision, 2011; Management and planning Organization, 2005), and particularly in the “map of health sector transformation” (MOHME, 2012).

Medical universities in Iran, as the largest organizational units in the health system, have an important role as trustee of health in the community, in production and expansion of health services (Rashidian, Jahanmehr, Pourreza, Majdzadeh, & Goudarzi, 2010). Monitoring and measuring of their deputy of health as the widest scope of the health system from the standpoint of volume of activities and the scope of services in the country, with respect to the possession of a large part of the health resources is particularly important. As well as conducting periodic evaluations of the performance of other sectors of the MOHME like research and education deputies, in recent years (Peykari et al., 2012), the ministry’s deputy of health also makes a priority for performance assessment and ranking of Health Deputies, with the aim of creating incentives to promote the performance of all medical universities. Therefore, providing a clear, logical and transparent conceptual framework for operating of mentioned objectives and priority are a key requirement. The main objective of this study was to design a conceptual framework, according to the policies and priorities of the MOHME to evaluate the performance of Health Deputies in medical universities and determine their share in the overall health impacts of the community.

## 2. Methods

The structure and process of the study were formed by a research group from Tehran University of Medical Sciences, MOHME, treatment and medical education and the National Health Research Institute. To achieve results - the conceptual framework -of this study, we used several tools and techniques. Each are explained as follows:

### 2.1 System Thinking

After the introduction of health system building blocks by WHO in 2007, using this method was recently proposed (De Savigny & Adam, 2009). System thinking is expressed as a deeper understanding of relationships, communication, behaviors and reactions among all constituent subsystems and elements of a system. Due to the complexity, and the nature of the continuous changes in the health system, by using system thinking we can focus on the relations between the components of the system, events, interactions and feedbacks between these components, very well (Adam & de Savigny, 2012). The structure of the Health Deputies, relationship between the main determinants of health, extraction of Results Chain Model and its communication and interactions between the various parts, are all achieved by this system view.

#### 2.1.1 Noting the Organizational Structure of Public Health and Primary Care

**Figure 1:**
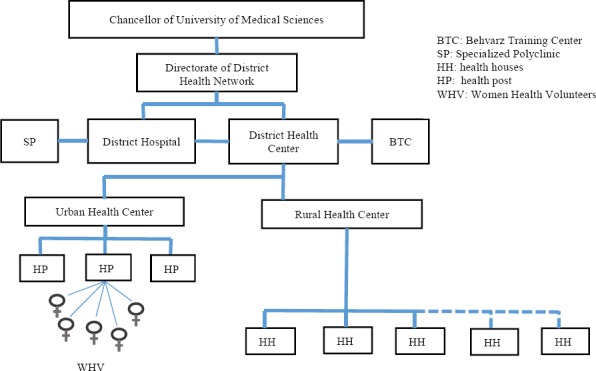
The structure of Health Network in Medical University; adapted from (Takian, 2011)

In the process of designing the conceptual framework, the comprehensive understanding of the components, communication and the various parts of the Health Deputies is necessary as the first step in this process. Reviews of their structure showed that every associated University of Medical Sciences to MOHME, has a Health Deputy with a characterized hierarchy and subset of health centers and networks (Figure 1). The MOHME of Iran has a centralized structure. In addition to its associated medical universities, it has several headquarter/staff deputies, with each of them monitoring and making policies on the similar and related deputies at the Universities of medical sciences. Combining the units and departments of Health Deputies at the University of Medical Sciences creates a composition similar to that of the Health Ministry Deputies, and each unit in addition to their respective universities, is also linked to a related unit in the MOHME.

Health Deputies, have two major sectors in their activities including public health and primary health care. In Iran, the size of the private sector in activities associated with prevention and primary health care in comparison with the public sector has been minimal, and almost all intervention and activities are done by the Government through the health networks (Figure 1). With regard to this issue, the private sector and its function has not been addressed in this study.

#### 2.1.2 Health System Components

**Figure 2:**
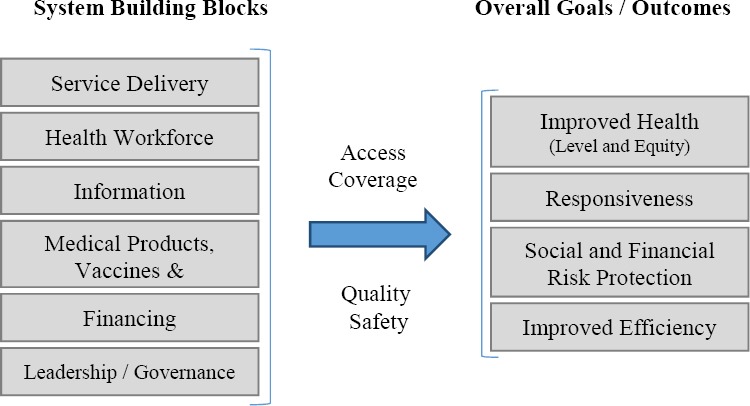
WHO overall health system goals and building blocks; adapted from (WHO, 2007)

Our other approach in designing the conceptual framework, would be agreement on the main components and factors affecting the performance of the health system and Health Deputies. The study conducted by the World Health Organization in 2007, aiming to determine the building blocks of the health system, was one of the best sources available on this topic (World Health Organization [WHO], 2007). Accepting the approach of the World Health Organization on the goals of the health system and introducing its building blocks, guides us well in various stages of study, including the process of choosing the indicators, selecting the components of the framework, communicating between them and the evaluation methods of the model. Improved health, responsiveness, financial and social risk protection and improving the efficiency are the overall health system goals, and leadership or governance, service delivery, human resources, information, financing and medical technologies and products are the building blocks and factors affecting the performance of the health system from the view of WHO (Figure 2).

The WHO’s definition of the health system is “all organizations, people and actions whose primary intent is to promote, restore or maintain health. This includes efforts to influence determinants of health as well as more direct health-improving activities (WHO, 2007). This is a good basis for determining a framework for the performance of the health system. The above definition shows that the health status of the people is not merely affected by the performance of the health system, and several factors, including a range of social and economic determinants affect people’s health status. Clearly, the control of all these factors is not available to Health Deputies and is not right that this section be responsible for things which it does not have complete control over (Murray & Frenk, 2000).

### 2.2 Literature Review

We searched the following sources: electronic databases, official websites of relevant national and international organizations, checked the reference lists of obtained studies, and searched general worldwide web search engines.

The PubMed, Scopus, Web of science, Google Scholar and two specialized databases of Persian language literature (IranMedex and Scientific Information Database) were searched with main terms and keywords such as: performance assessment, performance evaluation, performance measurement, health indicators, conceptual framework, assessment framework, health system performance, and monitoring and evaluation. The main searches were complemented by searches of organizations such as the WHO, World Bank, OECD, and the MOHME and Medical Education of Iran. The searches covered the period from 2000 to 2014. The searches were carried out with great sensitivity to extract all related and attainable studies. After the search, all obtained scientific resources, were reviewed by researchers and the scientific literatures related to the fields of study were extracted.

We looked for studies that developed conceptual frameworks, models or applications of public health and primary care monitoring and evaluation around the world and in Iran. In the reviews on the obtained studies, important issues like design components of conceptual frameworks and the process of choosing the desired indicators, were in the spotlight of the research group.

### 2.3 Collecting Key Stakeholder Views

To extract the knowledge of experts about performance monitoring and evaluation of Health Deputies, after designing a form with the title of structure and the properties of the interviewee, by the consultant of the research group, 15 skilled and experienced experts from different levels of the MOHME were selected and interviewed with. About the selection of experts, at the end of each interview, we asked experts to suggest people for the next interviews. The suggestions of the interviewee, were in most cases identical to those selected by the research group. Therefore, an interview guide was designed with18 open questions and a deep interview was carried out after taking the time from the experts. The length of each interview on average was about an hour. Interviews were performed between November 2012 and April 2013. The purpose of the interview and the questions of it, focused more on the Executive Protocol and the methods for study. Familiarizing the researcher with the main areas of performance in Health Deputies, the components of the conceptual framework and the target indicators for evaluation of the performance were other interesting topics. The results of the interviews in this study are not reflected, separately.

### 2.4 Identifying the Core Indicators for Monitoring and Evaluation

By use of the results from the qualitative part of the study as well as the review of other countries’ experiences, through several meetings carried out at different times by the research team, the indicators were selected after discussion about the goals of the health system and the strategies of the Health Deputies, as well as the information needs of different stakeholders, particularly the policy makers of MOHME. This process was carried out through collective agreements and decisions.

With the aim of considering all aspects of the performance of the Health Deputies, at the beginning of collection of indicators we did not consider any restrictions, and many sources like MDGs ‘MICs and WHO, national health indicators, indicators from various studies done by the MOHME such as Utilization and IrMIDHS were entered in the study. At this step, a number of 602 different indicators were identified. Then, to select appropriate and required indicators at a meeting attended by six experts of health system, all collected indicators in the previous step were reviewed. In this session a number of 250 indicators were selected. The selection procedure consisted of all of the indicators in the printed sheets, being given to experts and they being asked to select the appropriate indicators according to criteria including covering all performance areas in the Health Deputy, usefulness, availability and being SMART particular, relevant, achievable and measurable. Prior to the selection of indicators by the experts present at the meeting, the results of the qualitative study and comments and the approach outlined in interviews, as well as the results of reviews of the scientific literature and similar studies carried out in other countries, and in Iran were presented to them in a report. Furthermore, the list of indicators was presented at a national health observatory meeting conducted at the National Institute of Health Research. The meeting was attended by 30 people from different areas of the health system. As a result of these steps, 120 indicators were selected, divided into 11 categories: mortality, communicable and non-communicable diseases, maternal and child care, immunization, environmental health, professional health, health workforce, health facilities, social and economic, risk factors and health financing. Then the research team reviewed the list in iterative meetings in order to reduce the number of indicators to a limited number (WHO, 2011). During this process, indicators with similar focus were joined and a list of 51 core indicators associated with each area of the proposed conceptual framework were selected.

## 3. Results

### 3.1 Fundamental Questions in Performance Evaluation

Several important questions raised by various researchers that are responded to in the form of system thinking are a very good guide for designing the conceptual framework for performance monitoring and evaluation (Murray & Frenk, 2000; Papanicolas & Smith, 2010). These questions and topics include:

How will the proposed conceptual framework be related to the structure of the health system and the Health Deputies? What is the health system objectives and building blocks? And how will the proposed conceptual framework illustrate them? What is the performance concept and what are its influencing factors? What are the borders of the health system and the main determinants of health? And how does the proposed conceptual framework show these borders? What is the main purpose of the conceptual framework according to Health Deputies’ needs?

Although the above questions are mostly associated with the health system, but since Health Deputies are one of the important subsets of the health system in Iran, there is a strong relationship with the above questions and these Deputies. Furthermore, with regard to, a ‘whole to component’ approach- determining the performance of the Health Deputies among the different determinants of health- in this study, we try to provide an appropriate response to these questions with focus on the Health Deputies, while introducing details of the conceptual framework.

To design the conceptual framework with the above specifications and appropriate to the conditions and requirements of the Health Deputies, we reviewed most of the framework used at the international level (Arah, Klazinga, Delnoij, Ten Asbroek, & Custers, 2003; European Commission, 2013; Handler, Issel, & Turnock, 2001; Hogg, Rowan, Russell, Geneau, & Muldoon, 2008; Canadian Institute for Health Information [CIHI], 2012; Kelley & Hurst, 2006; Murray & Frenk, 2000; WHO, 2007; Papanicolas & Smith, 2010; Ten Asbroek et al., 2004; Wong et al., 2010). Using the experience of other countries, along with health policies and priorities in Iran, led to the design of a framework based on the objectives of the study. The proposed conceptual framework is described as follows.

### 3.2 Proposed Conceptual Framework

**Figure 3:**
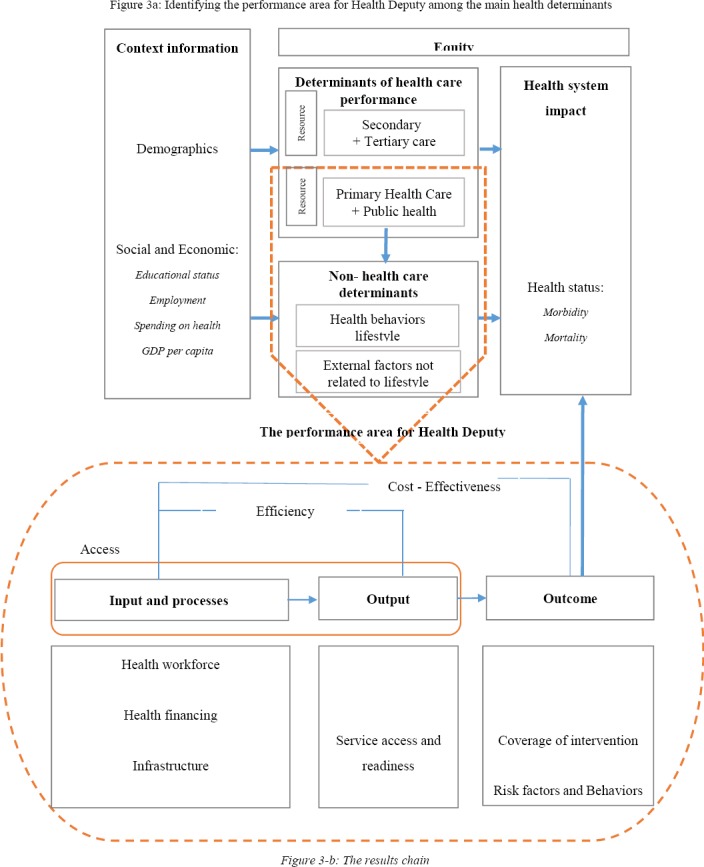
The proposed Conceptual Framework for performance evaluation of Health Deputies in Iran

Due to the multiplicity and complexity of the relationships in health determinants, as previously mentioned, one of our approaches in the design of the framework, was to show the main determinants of health and determine the role and share of the Health Deputies among them. There are several studies, which determine the determinants of health, but the study by the social protection Committee (SPC), related to the European Commission, with a deeper vision, has addressed the appropriate boundaries through the introduction of the Joint Assessment Framework (JAF) (European Commission, 2013). We used the results of this study, while outlining the main determinants of health, and have determined the boundaries of the health system in Iran. Then while taking into mind Iran’s health system, we specified the areas related to the performance of Health Deputies in the JAF model. After this step, the specified areas that had been transferred to the WHO proposed framework, were called the Results Chain (WHO, 2011). In this way we have introduced and proposed a new model, which through describing the relations between its various components, provides monitoring and evaluation of the performance of Health Deputies. The proposed model shows the contribution and the role of Health Deputies on health impacts (Figure 3).

#### 3.2.1 Determine the Performance Area for Health Deputy

Based on the results of an SPC study and other research like WHO and OECD, the boundaries of the health system can be divided into two categories: determinants of direct performance of the healthcare system and topics that are out of the health system, or in other words, non-healthcare system determinants. The overall health impacts in the proposed model are determined by these boundaries. The first boundary was shown with indicators, which shows that if people need health care, they can receive it with good quality through health system interventions (Figure 3a).

The overall impacts of health in this model were the main results expected from a healthcare system that shows the health status, including mortality and morbidity in the population. These indicators can be measured by things other than the health system, and as previously mentioned they are affected by several determinants. The most important indicators chosen for this section have been described in Table 1.

**Table 1:** The list of core indicators for monitoring and evaluation of proposed framework

No	Indicator	Type of indicator	Indicator domain	Data source
1	Urban health centers	Input and process	Infrastructure	Deputy of health-MOHME-2011
2	Rural health centers	Input and process	Infrastructure	Deputy of health-MOHME-2011
3	Active health house	Input and process	Infrastructure	Deputy of health-MOHME-2011
4	Rural health posts	Input and process	Infrastructure	Deputy of health-MOHME-2011
5	Urban health posts	Input and process	Infrastructure	Deputy of health-MOHME-2011
6	Number of Family Physicians	Input and process	Health workforce	Deputy of health-MOHME-2011
7	Number of Midwives	Input and process	Health workforce	Deputy of health-MOHME-2011
8	Number of employed behvarz	Input and process	Health workforce	Deputy of health-MOHME-2011
9	Percent of deaths that are registered (births registered)	Input and process	Information	Deputy of health-MOHME-2011
10	General government expenditure on health as % of general government expenditure	Input and process	Health financing	Deputy of health-MOHME-2011
11	Treatment success rate (TB DOTS)	Output	Service quality and safety	Health profile indicators in Iran-2011
12	Delivery ratio by cesarean section	Output	Service quality and safety	IrMIDHS*-2010
13	Disposal of Waste children	Output	Service quality and safety	IrMIDHS-2010
14	Desirability of removing soda bread samples	Output	Service quality and safety	Health profile indicators in Iran-2011
15	Access to sanitary toilets in rural	Output	Service access	Health profile indicators in Iran-2011
16	Use of optimum toilette system by household members (%)	Output	Service access	IrMIDHS-2010
17	Infants weighed at birth	Output	Service access	IrMIDHS-2010
18	Access to safe drinking water in rural areas	Output	Service access	Health profile indicators in Iran-2011
19	Use of drinking water from optimized sources	Output	Service access	IrMIDHS-2010
20	Refined Iodized salt in public places	Output	Service access	Health profile indicators in Iran-2011
21	Percentage of employees covered by employment examinations	Output	Service access	Health profile indicators in Iran-2011
22	The prevalence of hypertension	Outcome	Risk factor and behaviors	Health profile indicators in Iran-2011
23	Percent of obese people (BMI≥30) - Women	Outcome	Risk factor and behaviors	NCDRFS**-2009
24	Percent of obese people (BMI≥30) - men	Outcome	Risk factor and behaviors	NCDRFS -2009
25	The prevalence of severely underweight children under 5 years	Outcome	Risk factor and behaviors	IrMIDHS-2010
26	The prevalence of severe underweight in children under 5 years	Outcome	Risk factor and behaviors	IrMIDHS-2010
27	The prevalence of severe stunting in children under 5 years	Outcome	Risk factor and behaviors	IrMIDHS-2010
28	The prevalence of infants with low birth weight (LBW)	Outcome	Risk factor and behaviors	IrMIDHS-2010
29	Percentage of people who are daily smokers - Women	Outcome	Risk factor and behaviors	NCDRFS -2009
30	Percentage of people who are daily smokers - men	Outcome	Risk factor and behaviors	NCDRFS -2009
31	Prevalence of low physical activity	Outcome	Risk factor and behaviors	Health profile indicators in Iran-2011
32	Measles vaccine coverage	Outcome	Coverage of intervention	Health profile indicators in Iran-2011
33	Polio vaccine coverage (third time)	Outcome	Coverage of intervention	Health profile indicators in Iran-2011
34	Prenatal care coverage (at least twice care)	Outcome	Coverage of intervention	IrMIDHS-2010
35	Postnatal care coverage (at least one cares)	Outcome	Coverage of intervention	IrMIDHS-2010
36	Deliveries in the presence of trained health care providers (%)	Outcome	Coverage of intervention	IrMIDHS-2010
37	Deliveries at health centers (public and private)	Outcome	Coverage of intervention	IrMIDHS-2010
38	Prenatal care is covered by the educated or trained caregivers	Outcome	Coverage of intervention	IrMIDHS-2010
39	Coverage/percentage of contraceptive users	Outcome	Coverage of intervention	IrMIDHS-2010
40	Percentage of children under 5 years with diarrhea	Outcome	Coverage of intervention	IrMIDHS-2010
41	Rates of exclusive breast feeding up to 6 months	Outcome	Coverage of intervention	IrMIDHS-2010
42	Infant mortality rate (per thousand live births)	Impact	Health status	Mortality profile in 29 provinces during 2005-2010
43	Under 5 mortality rate(per thousand live births)	Impact	Health status	Mortality profile in 29 provinces during 2005-2010
44	Total fertility rate	Impact	Health status	National Organization for Civil Registration-2009
45	The incidence of TB(positive smear)	Impact	Health status	Health profile indicators in Iran-2011
46	The incidence of measles	Impact	Health status	Health profile indicators in Iran-2011
47	Cases of neonatal tetanus	Impact	Health status	Health profile indicators in Iran-2011
48	The rate of the population is covered by Medical Universities in different age groups	Demographic	Demographic	Deputy of health-MOH-2010
49	Education(Years of schooling)	Social and economic	Social and economic	NCDRC***-2010
50	Urbanization(Male/Female)	Social and economic	Social and economic	NCDRC-2010
51	Wealth index	Social and economic	Social and economic	NCDRC-2010

* Islamic Republic of Iran’s Multiple Indicator Demographic and Health Survey

** Iran Non-Communicable Disease Risk Factor Surveillance

*** Non-Communicable Disease Research Center

The second boundary was shown with determinants outside the healthcare system including risk factors and factors related to lifestyle and behavior of individuals as well as factors that are non- related to lifestyle, such as environmental factors (Figure 3-a). These factors have a good potential for prevention activities, including education and health promotion in order to improve the health of the population. Due to the notable increase of non-communicable diseases in recent years and the unfavorable status of Iran between the 20 nations in the region (Shahraz et al., 2014), monitoring and control of risk factors for these diseases has now become one of the main priorities of the health system, especially in Health Deputies. It can be said that a large part of the difference in the community health indicators is not due to differences in health care but rather is indebted to the amount of success in health promotion and disease prevention activities in Health Deputies (European Commission, 2013). Environmental factors related to the second category of health determinants were not entered in the model.

Furthermore, the proposed model shows a range of determinants and socio-economic backgrounds, including occupational status, education, demographic information, poverty and social exclusion, health expenditure and per capita income that are outside of the health system boundaries, while having effects on both categories of determinants related to the performance of the health system and non-healthcare system determinants, and are therefore associated with overall health indicators (Figure 3-a). The indicators associated with this area have also been described in Table 1.

Due to the difference between the Treatment Deputy and the Health Deputy in the structure of the MOHME in Iran-both in terms of planning and management, and resources and input variables- we have broken down the health system performance in the proposed model into two areas, the first being specialized and subspecialty medical services related to the secondary and tertiary levels of referral and the second being public health and primary health care services related to the first level of referral in the health system (Figure 3-a). As mentioned earlier, due to the potential effect of determinants related to public health activities on behavior and lifestyle in comparison with other determinants of health (European Commission, 2013), and the priority of effects on risk-factors and reducing them as a strategy in the current Health Deputies in the MOHME, these two areas including both public health and primary health care services alongside behavior and lifestyle determinants, have been chosen as the main areas used for performance evaluation of Health Deputies (Figure 3-a).

According to a recent description, performance measurement and evaluation of the Health Deputies do not mean evaluation of all health systems, and the Health Deputies’ role should be seen alongside the performance of other determinants of the health system. In the next section, we will show the performance of the Health Deputies in the form of the Results Chain model.

#### 3.2.2 Results Chain: Monitoring and Evaluation of Performance in the Health Deputy

**Figure 4:**
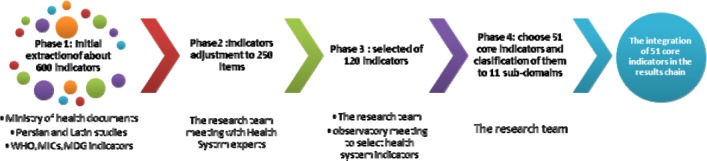
4 phases of identifying the core indicators for performance evaluation of Health Deputies

The results chain as a framework for monitoring and performance evaluation in Health Deputies, is shown in Figure 3-b. This chain consists of three main areas of indicators: inputs and processes, outputs and outcomes. Chain of results shows how to reflect the input and process (such as manpower and equipment) into output and outcome indicators (such as child and maternal care and access to safe water). As previously mentioned, our main goal was focused on the performance of the Health Deputies, so impact indicators due to the influence of other determinants of health were not entered in the results chain. These indicators are located within the context of all determinants of health in Figure 3-a. Of course; according to the previous description, results chain model as the Health Deputies’ performance area is only one of the main factors that affects impact indicators in the health system, and these are specified in Figure 3. In the results chain model inputs, processes and outputs reflected the capacity of Health Deputy. Furthermore, inputs and outcomes were the results of investment and in fact, represented the performance of the Health Deputy (WHO, 2011). As can be seen in Figure 3-b, each main area has several sub-domains of indicators that have also been mentioned, following the main area. Each sub-domain consists of several core indicators. Table 1 shows the core selected indicators that are broken down to the different areas in the results chain. Among the 602 obtained indicators, through the course of several steps (Figure 4) a final number of 51 were selected for monitoring and evaluation of the proposed framework (Table 1). In the method section we described how indicators were chosen.

Some of the core indicators that were related to more important running vertical health programs, were put in the final list, to show their effects on outcomes of the community’s health. In the proposed model, and in the selection of indicators based on the recommendation of the World Health Organization, we have tried to cover all age groups from childhood to adulthood.

#### 3.2.3 Performance dimensions and the Operational Domains for Evaluation of Health Deputies

When designing a conceptual framework, one of the main topics taken into consideration in various studies, was the different dimensions related to performance. Potentially, these dimensions describe health system performance and act as levers for health improvement (CIHI, 2012). Actually, dimensions of health system performance in any country are the ones that are definable, measurable and applicable in practice. Furthermore, they must be attributable to the Health System functions in accordance with its goals (Kelley & Hurst, 2006). Studies conducted in other countries, indicated various dimensions of performance in their introduced framework (Table 2). According to the objectives of each study, these dimensions are different in other countries. For example, among the studies mentioned in Table 2, the study related to CIHI carried out in Canada had almost all the dimensions of performance, and is a fairly comprehensive study in this field. Some of the introduced dimensions in different frameworks were operational in the evaluation system of performance in other countries, while others had remained in the ‘definition and proposition’ stage (Kelley & Hurst, 2006). The most important dimensions that we proposed according to the above definition include efficiency, effectiveness, equity, access to health services and improving the health status. In fact, the proposed framework of this study would include more repeated dimensions in other countries and have high similarity to the World Health Organization framework (Table 2).

**Table 2:** Comparison of performance dimensions in the proposed framework for Iran with others

Dimensions	Commonwealth Fund (2006)	WHO (Handler et al., 2001)	OECD (Hurst et al., 2001)	CIHI (Canada) (CIHI, 2012)	OECD (Kelley & Hurst, 2006)	Proposed framework for Iran
Accessibility	✓		✓	✓		✓
Comprehensiveness				✓		
Integration				✓		
Appropriateness of care	✓			✓		
Safety	✓			✓	✓	
Effectiveness	✓	✓		✓	✓	✓
Responsiveness	✓	✓	✓	✓	✓	
Expenditure or Cost		✓	✓			
Efficiency	✓	✓	✓	✓		✓
Health status improvement	✓	✓	✓	✓		✓
Equity	✓	✓	✓	✓		✓
Innovation	✓			✓		

These dimensions were suggested according to the goals and strategies of the health system and Health Deputies in Iran and review of the WHO research and the experiences of other countries. We relied on the WHO study for the definition of each of these (Handler et al., 2001). By use of these dimensions, and based on them, we introduced several qualitative and quantitative ways for analysis of information, measurement of performance and comparing all Deputies with each other in the monitoring and evaluation system, including the following items:

3.2.3.1 Progress Towards the Goals of the National Health System

In this way the extent of the achievement of the Health Deputies’ predetermined goals will be monitored for each of the core indicators. For example, what percentage of the goals in the tuberculosis care program were achieved in the previous year? Due to lack of strategic programs, the majority of Health Deputies are not in good condition in this field.

3.2.3.2 Measurement of Efficiency

A monitoring and evaluation system should measure the amount of health benefits and results that had been created for the community, compared to the resources used. Increasing efficiency is one of the main objectives of implementation of the monitoring and evaluation system. The efficiency will focus on the ratio between output and input indicators (Figure 3b).

3.2.3.3 Accessibility

Access to health care has different aspects including physical and financial access, particularly. Measuring the amount of access to the various health services, in the input and output area, and, in particular, its physical aspects as an important dimension of performance, makes it possible to compare Health Deputies at the University level (Figure 3b). Due first-level services being relatively free of charge in Iran, financial access is not very notable.

*3.2.3.4 Equity*

Access and equity dimensions are closely related in the health system. Measuring progress on issues related to the distribution of resources and the achieved result is very important. Reviews of issues related to equity in provincial and university levels were of interest to most related managers (Figure 3a).

*3.2.3.5 Qualitative Assessment*

For a comparison of the changes in the Government’s macro-policy and management as well as management and leadership changes at different levels of the MOHME, conducting qualitative studies on the monitoring and evaluation system is a necessity. Analysis of the information obtained from qualitative studies along with quantitative results, will be the basis for the next plans and policy making of the Health Deputies.

*3.2.3.6 Benchmarking*

There are various types of benchmarking, use of which depends on cases such as the levels of comparison (between provincial, national and international), focus on the areas of measurement (access or coverage) and the levels of information usage. Furthermore, the benchmarking procedures are different. Based on these procedures Health Deputies performance can be compared to each other. For example, comparison can be based on: the best performance among the Health Deputies, the level of achievements in a national or international goal in relation to one or more specific indicators or comparisons based on the past performance of the Health Deputies in a period of time.

*3.2.3.7 Cost-Effectiveness*

Managers and policy makers used cost-effectiveness analysis as a tool for evaluation and enhancing the performance of the health system. Due to lack of resources, cost-effectiveness analysis can be used for priority setting of interventions and also optimizing the resource allocation in the Health Deputies. Of course, in order to carry out a cost- effectiveness analysis, we first need to determine the effectiveness of different interventions, risk factors and burden of diseases (Figure 3b).

## 4. Discussion

This study illustrates a conceptual framework of performance for the Health Deputy in Universities of Medical Sciences by showing their performance area among other determinants of health, and introduction of the results chain for them.

One of the strengths of this study is introduction of several dimensions for performance that make it possible for us to evaluate the performance of Health deputies and compare them in different ways. For example if managers want to compare all Deputies using only their efficiency, they can do this using the results chain. Also, for optimum planning and policy making in universities we proposed to conduct a qualitative study as well as quantitative methods for identifying the changes in the health system.

Another strong point of the study is the consultation it has done with a wide range of different organizations and experts, most of which were major stakeholders in the performance evaluation of the Health Deputies in Iran. The main purpose of this work was to design a fairly acceptable and applicable framework in practice. However, we believe the proposed model has its flaws, and by revision and interaction with various stakeholders can be made more complete, and have increased value.

With regard to the existence of the very large number of running vertical programs in Health Deputies, related to different diseases and health problems (such as the tuberculosis care program and the diabetes prevention and control program), following the interviews with experts, some of them recommended that the proposed framework for monitoring and evaluation should be based on these programs, In other words, they said it is necessary for all health programs designed and delegated to Health Deputies by the MOHME to be evaluated. For each of the vertical health programs, there are hundreds of indicators, from national and international resources. Collecting information for all of these indicators is expensive and time consuming. The interpretation of this data is also difficult and there will be a lot of concerns over the quality of data and the relation between collected data. So one of the main challenges for monitoring and evaluation of the Health Deputies, is the selection of core indicators, which are able to monitor the movement towards the desired objectives in a targeted and efficient way (WHO, 2011). Therefore in the proposed framework in this study, we did not enter all indicators of vertical programs, but rather chose the more important ones (For example, in a final list, indicators like “the prevalence of hypertension” and” percent of obese people (BMI≥30)” are related to the fight against non-communicable diseases program or “treatment success rate (TB DOTS)” related to the program of fight against tuberculosis disease). One of the other advantages to this model is that the results chain, in addition to the province or national-level can be also used for monitoring and evaluation of one specific vertical program, for example, the oral and dental health improvement program, because each of these programs have their particular operational plans and strategies, and the principles of the results chain can be applied in their case.

In designing the proposed framework, we were faced with a few major challenges. All parts of the health system were not our main goal in this study, and we had to determine and separate the performance areas for the Health Deputies from the healthcare system. According to the MOHME structure, this subject posed as our first challenge. Although the MOHME in Iran currently has a breakdown structure, and its Health and treatment deputies are separated, this separation is not really true, since activities and interventions related to the family physician and primary health care are still within the scope of Health Deputy Responsibilities. In this regard, Iran’s health system in the past decade has changed its structure several times, at one time merging these two deputies and at other times separating them. This issue was not solely Iran’s challenge. Furthermore, the distinction between the health activities and medical services and defining their relations with the health of population, continues to remain as a challenge for other countries (Arah et al., 2003). We have to overcome this challenge by using different levels of services and the referral system approach in the health system.

In the health system of Iran there is a referral system with three levels of services. The main focus of this study is on first-level services. To determine the performance area in Health Deputies, in the proposed model, medical and hospital services related to the second and third-level of the referral system were separated from first-level services.

The second challenge and our main concern was determining the extent of the accountability and the role of the Health Deputies on the overall health impacts in the community. Our review showed that this concern also existed in other studies that worked on health performance evaluation (Murray & Frenk, 2000). The main question was whether the Health Deputies were solely responsible for their actions within the organization or whether they should be accountable for broad health determinants outside of their performance area. Perhaps it is not fair that the Health Deputies be accountable for results that are not totally in their control. Especially since a lot of the policy making and planning that aims to solve community problems, carried out by the MOHME, is done so without the cooperation or consultation of relevant deputies.

The Health Deputies can affect overall health impacts (such as under five-year mortality) through determinants that are out of the boundary of the healthcare system, in addition to their direct responsibilities, therefore increasing their achievements in the health sector and in this way validating the extent of its accountability. To fix this concern, based on other studies in this field (European Commission, 2013; Kelley & Hurst, 2006), we broke down the main determinants of health, and by explaining the relations between them, determined the performance area for the Health Deputies, among the various determinants of health (Figure 3a).

The last challenge was related to the concentration of policy making in the MOHME. In Iran, the universities are the executives of the MOHME policies and policy making cannot be done by them alone. Any evaluation of the performance in this system depends on the extent of the success in subset units, in achieving the goals of these policies. The results and outcomes due to health functions of universities, in fact, were the endpoint of the policies and programs made by the MOHME. It can be said that due to lack of complete independence in universities in this structure, the MOHME, but not the Health Deputies, is responsible for the large part of the results of any performance evaluation. Any action aiming to increase the powers and authorities of the Health Deputy and reduce the concentration of policymaking in the ministry, would affect the results of monitoring and evaluation. Considering the differences in needs and the speed of transformation in the epidemiological profile of the country, it is necessary that a greater part of authority for planning be transferred to the Health Deputies, so that is becomes possible to focus on local needs. This may need a major investment on improving the information system and management capacity until the subset units become able to assess their needs and carry out planning.

The proposed framework can be used as a basis for evidence-based policy-making in different levels of the health system. The optimal allocation of resources, proper use of existing facilities, monitoring the rate of indicators’ improvement in the results chain, the creation of healthy competition among Health Deputies through their annual comparison, providing appropriate feedback to health service providers and ultimately improving the performance of the Health Deputies were the other advantages of correct implementation of the proposed framework. Furthermore, applying this framework can be an important step in supporting strategic planning in the Health Deputies and a valuable tool in increasing the accountability of the health system by providing regular performance reports.

Also, the flow of information in the Health Deputies, is only from service providers to organizations which collect data, particularly the MOHME and therefore not enough feedback is given to those that registered the data or provided the services. Providing feedback to primary health care and public health service providers is essential for the following reasons: to inform them about their work dimensions and the results of their interventions, to empower them for good and timely reactions to their performance and finally to change their behavior for proper implementation of the monitoring and evaluation system (WHO, 2011).

## 5. Conclusion

This study was done with the aim of designing a conceptual framework to evaluate the performance of Health Deputies at Universities of Medical Sciences, and determine their share in the overall health impacts among the major determinants of health in Iran. Therefore it has tried to determine the performance area, introduce a chain of results, and identify the several layers of indicators and the different aspects of performance evaluation, thereby showing how Health Deputies can achieve the best results, and how we can observe the best consequences of health in the community.

Without a doubt, full implementation of the proposed framework to measure and evaluate performance needs to determine assignments and accept the roles of stakeholders, particularly in the MOHME and Health Deputies at the Universities of Medical Sciences. Having the relative commitment of these organizations is essential in order to achieve the desired results and provide the performance report.
